# Circulating Human Neonatal Naïve B Cells are Deficient in CD73 Impairing Purine Salvage

**DOI:** 10.3389/fimmu.2016.00121

**Published:** 2016-03-30

**Authors:** Matthew Aaron Pettengill, Ofer Levy

**Affiliations:** ^1^Division of Infectious Diseases, Department of Medicine, Boston Children’s Hospital, Boston, MA, USA; ^2^Harvard Medical School, Boston, MA, USA; ^3^Precision Vaccines Program, Boston Children’s Hospital, Boston, MA, USA

**Keywords:** B cells, purine, purinergic, adenosine, CD73, neonatal, salvage

## Abstract

**Background:**

Extracellular purines, in particular adenosine (Ado) and adenosine-triphosphate, are critical immunoregulatory molecules. Expression and activity of purine ecto-enzymes on B cells in neonatal and adult blood may influence their function and has been incompletely characterized.

**Methods:**

Mononuclear cells were isolated from human neonatal (cord blood) or adult (peripheral blood) subjects and evaluated directly by flow cytometry for expression of purine ecto-enzymes. Additionally, B cell subsets were isolated from mononuclear cell fractions by fluorescence-activated cell sorting and gene transcription of purine ecto-enzymes (CD39 and CD73), Ado deaminase (ADA1), purine nucleoside phosphorylase, and select purine receptors (A2a) were evaluated by reverse transcription followed by qRT-PCR. Immuno-magnetic-bead isolated naïve B cells were evaluated for enzymatic activity by incubation with radio-labeled purines followed by thin-layer chromatography, and subsequent B cell Ado acquisition was evaluated by liquid scintillation quantitation of radio-labeled Ado uptake.

**Results:**

Relative to their adult counterparts, neonatal circulating naïve B cells were markedly and selectively deficient in CD73 as observed by gene transcription, surface protein expression, and enzyme activity. Neonatal naïve B cell deficiency of CD73 expression significantly impaired their capacity to acquire extracellular purines for purine salvage.

**Conclusion:**

Human neonatal circulating naïve B cells are selectively deficient in CD73, impairing extracellular purine acquisition and potentially contributing to impaired B cell responses in early life.

## Introduction

B cells develop from pluripotent precursors in the bone marrow, enter circulation as mature naïve B cells, and eventually traffic through lymphoid and non-lymphoid tissues in search of antigen. Activation by specific antigen along with helper T cells in lymphoid tissue can trigger further maturation (1), clonal proliferation, changes in the immunoglobulin (Ig) locus, and antibody (Ab) production and secretion. While Ab production plays a critical role in protection from infectious agents, other functions of B cells, including cytokine production and antigen presentation to T cells (2, 3), help shape immunity as well. Neonates are particularly susceptible to infection, in part reflecting distinct immunity in early life (4). Among the differences in immunity in early life, neonatal B cell function is distinct from that of adults (5, 6), but the underlying mechanisms are incompletely characterized.

Of note, purine metabolism plays an important role in regulating many B cell functions. Extracellular purine di- and tri-phosphates are dephosphorylated by CD39 (ENTPD1) and other related ecto-enzymes generating adenosine mono-phosphate (AMP), while CD73 (ecto-5′ nucleotidase) dephosphorylates AMP generating adenosine (Ado), an immunoregulatory molecule that can be further metabolized by Ado deaminase (7). Genetic deficiency of Ado deaminase (ADA1) or purine nucleoside phosphorylase (PNP) disrupts intracellular purine metabolism and leads to severe-combined immunodeficiency (SCID) with insufficiency of functional lymphocytes (8, 9). Additionally, purine nucleoside analogs that disrupt intracellular purine salvage inhibit B cell proliferative responses (10). B cells from patients with common variable immunodeficiency (CVID), characterized by limited Ab production and frequent infections, have low expression of CD73 and, thus, impaired metabolism of extracellular purines (11). Additionally, in a given human adult individual, subsets of B cells with reduced CD73 expression have a more limited capacity to undergo class-switch recombination *in vitro* (11).

Purine enzyme expression, including CD73, is regulated during lymphocyte maturation. In mice, CD73 is expressed primarily on B cells that have undergone class-switch recombination (12), and is a marker of memory (13, 14). Murine germinal center B cells express increasing levels of CD73, whereas plasmablasts and bone marrow plasma cells have little to no CD73 expression (15). In humans, AMPase activity was lower on circulating total B cells in newborn cord than adult blood (16), with neonatal B cell AMPase activity reaching adult levels by 6–12 months of age (17). However, these studies did not clarify if the differences were due to higher activity in adult cells as a result of greater expression of CD73 [or tissue non-specific alkaline phosphatase (TNAP)] on memory B cells, which are present at significantly lower levels in newborns.

To gain insight into the ontogeny of purine metabolism on human B cells, we sought to more fully characterize the expression of purine enzymes on circulating neonatal and adult B cell subsets, and to evaluate the impact of CD73 expression on B cell acquisition of extracellular purines. We found that circulating human neonatal B cells are deficient in CD73 expression and function, potentially contributing to impaired B cell responses in early life.

## Materials and Methods

### Blood Collection

Peripheral blood was collected after informed consent from healthy adult volunteers according to Boston Children’s Hospital Institutional Review Board-approved protocols (Boston, MA, USA; mean age 31.8 years, range 23–40 years), and newborn cord blood (mean gestational age 39.1 weeks, range 37.4–41.1 weeks) was collected immediately after elective cesarean section delivery (epidural anesthesia) of the placenta. Births to HIV-positive or febrile mothers were excluded. Human experimentation guidelines of the US Department of Health and Human Services, the Brigham and Women’s Hospital, Beth Israel Medical Center, and Boston Children’s Hospital were observed, following protocols approved by the local institutional review boards. Number of repeats (*N*) indicates number of unique human subjects evaluated per experimental procedure, no subject was studied more than once in each of the different experiments. Blood was collected into syringes containing a final concentration of 20 U/mL heparin (Sagent Pharmaceuticals; Schaumberg, IL, USA) and was used within 2 h of collection.

### Mononuclear Cell Isolation and Magnetic Bead Naïve B Cell Isolation

Peripheral blood mononuclear cells (PBMC) or cord blood mononuclear cells (CBMC) were isolated from heparinized whole blood by Lymphoprep (Accurate Chemical; Westbury, NY, USA) density gradient centrifugation. Non-naïve B cells (CD27^+^ B cells, T cells, NK cells, monocytes, dendritic cells, granulocytes, platelets, and erythroid cells) were labeled with a cocktail of biotinylated CD2, CD14, CD16, CD27, CD36, CD43, and CD235a Abs and magnetically labeled with Anti-Biotin MicroBeads for depletion (Naïve B Cell Isolation Kit II, human, Miltenyi Biotec, Auburn, CA, USA). To improve purity, unlabeled flow-through cells were subsequently labeled with CD19 microbeads (Miltenyi Biotec) for isolation of highly pure naïve B cell populations. All assayed samples had naïve B cell purities of >90% [mean CD20^+^IgD^+^ purity: 96% adults and 95% for newborns, purity was evaluated by flow cytometry as described below but with CD20 (APC-Cy7) antibodies instead of CD19 targeting antibodies]. Magnetic bead-isolated naïve B cells were utilized in enzyme activity and purine uptake assays.

### Flow Cytometry

**Figure 1:**
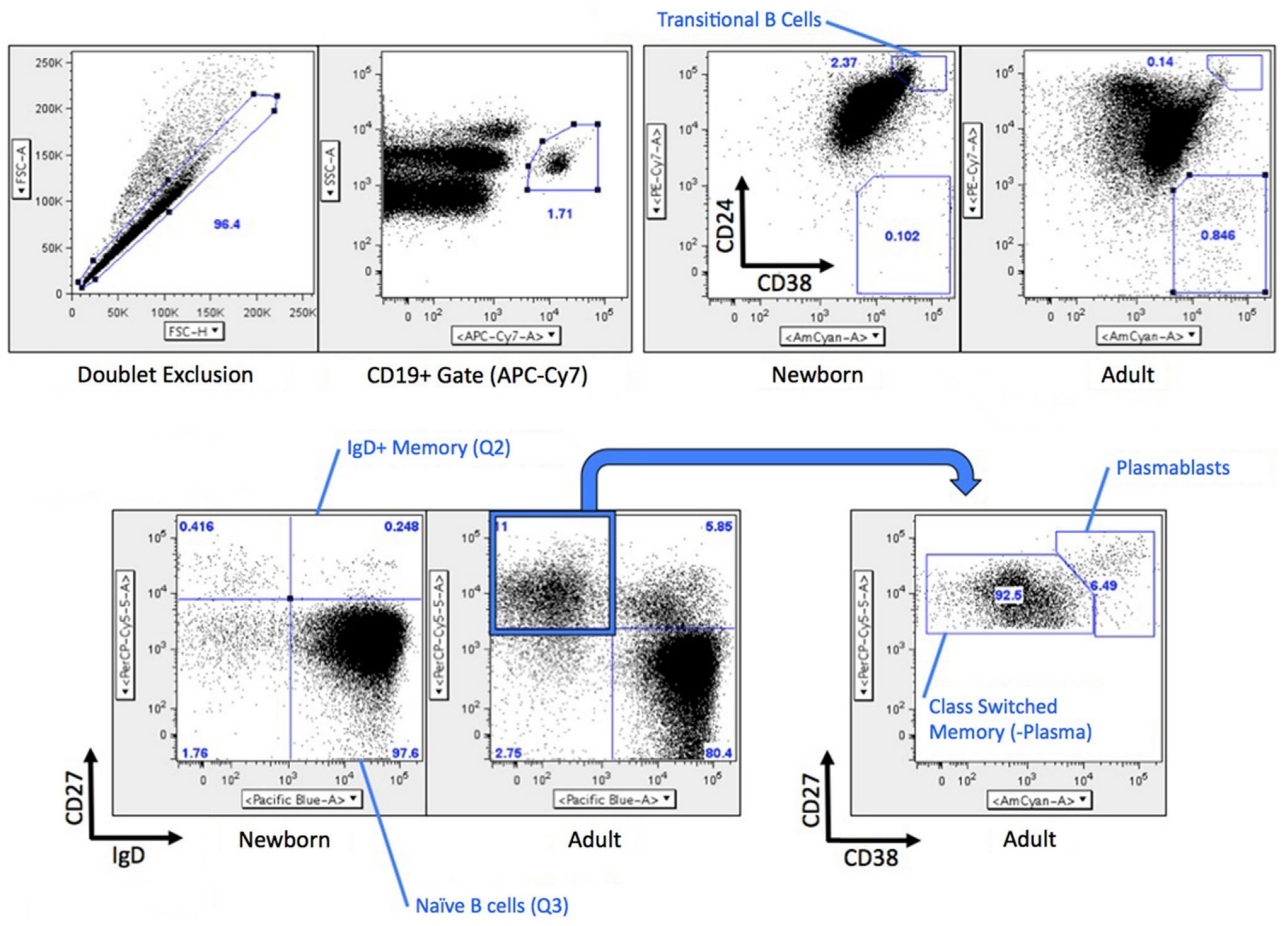
**Gating strategy for B cell flow cytometry**. Briefly, CD19^+^ B cells were analyzed for differential expression CD24/CD38 (transitional naïve B cells CD38^++^CD24^++^) or IgD/CD27 (naïve B cells IgD^+^CD27^−^, IgD^+^ memory B cells IgD^+^CD27^+^, switched memory B cells and plasma cells are both IgD^−^CD27^+^). CD19^+^CD27^+^IgD^−^ cells were further analyzed by CD38 expression (switched memory B cells CD38^±^, plasmablasts CD38^++^). A similar gating strategy was used for fluorescence-activated cell sorting (FACS) with the exception that each population was a more conservative independent gate inside of each quadrant for the CD27 vs. IgD parameters Transitional B cells were not flow sorted.

Mononuclear cells were stained with Abs (BD Biosciences, Frederick, MD, USA) targeting CD19 (APC-Cy7), CD24 (PE-Cy7), CD27 (PerCP-Cy5.5), CD38 (BV-605), IgD (BV-421), CD39 (FITC), and CD73 (APC). Stained cells were stained in PBS for 30 min at 4°C. Cells were then washed with PBS before fixation with 4% paraformaldehyde (Alfa Aesar, Ward Hill, MA, USA). Fluorescent intensities were detected using an LSR Fortessa flow cytometer (BD Biosciences), and gating strategies depicted in Figure 1 were based on previous characterization of B cell subsets from human pediatric and adult subjects (18). Compensation was performed in DIVA software (BD Biosciences) and fluorescent intensities were analyzed using Flowjo software version 10 (Tree Star Inc., Ashland, OR, USA).

### Fluorescence-Activated Cell Sorting

Fluorescence-activated cell sorting (FACS) was utilized to acquire highly pure B cell subsets for analysis of mRNA expression. Mononuclear cells were stained with Abs targeting CD19 (APC-Cy7), CD24 (PE-Cy7), CD27 (PerCP-Cy5.5), CD38 (BV-605), and IgD (BV-421). Cells were sorted using a FACSAria II cell sorter (BD Biosciences) utilizing the gating strategy described above (Figure 1). In brief, B cells were CD19+, and subsets were naïve B cells (IgD^+^CD27^−^), IgD^+^ memory (IgD^+^CD27^+^), class-switched memory (IgD^−^CD27^+^CD38^±^), and plasmablasts (IgD^−^CD27^+^CD38^++^). B cell subpopulations were sorted into tubes containing RPMI 1640 media (Invitrogen, Carlsbad, CA, USA) supplemented with 10% Fetal Bovine Serum (FBS, HyClone, VWR; Radnor, PA, USA), centrifuged at 500× *g* for 10 min, and pellets resuspended in Buffer RLT (Qiagen GmbH; Hilden, Germany) for RNA isolation.

### RNA Purification and cDNA Synthesis

Total RNA was isolated from sorted B cell subpopulations using the RNeasy Mini Kit with RNase-free DNase treatment (Qiagen GmbH; Hilden, Germany). Up to 300 ng of mRNA was reverse-transcribed to cDNA using the RT2 First-strand Kit (SABiosciences, Frederick, MD, USA), according to the manufacturer’s instructions.

### qRT-PCR

Expression levels of selected genes were assessed by qRT-PCR analysis using an ABI 7300 real-time PCR system machine and software (Applied Biosystems; Foster City, CA, USA). The baseline adjustment method of the ABI 7300 software was used to determine the cycle threshold (Ct) in each reaction. A melting curve was constructed for each primer pair to verify the presence of one amplicon-specific peak and the absence of primer dimerization. All samples were amplified in triplicates and the mean was used for further analysis. Relative expression of target gene mRNA was compared to that of the “housekeeping” gene β-actin (actB) using the ΔΔ-Ct method.

### Primer Sequences

ADA1, Forward 5′-TGGTTTCAGGCTTGATGGA-3′, Reverse 5′-GGCAGAGACCCACCGAG-3′; PNP, Forward 5′-GAAGCCATTCTCAGTGTTCT-3′, Reverse 5′-TTGCTCAGTTCAGCATAGCG-3′; CD73, Forward 5′-TTTGGCCTCTTTGAGGAGTG-3′, Reverse 5′-GGCACTATCTGGTTCACCGT-3′; CD39, Forward 5′-CCCACAGCAAGCAAAGCTA-3′, Reverse 5′-GGGGAAAGACGAGGAAAGAG-3′; TNAP, Forward 5′-ACCTGCTTTATCCCTGGAGC-3′, Reverse 5′-CTTGTGCCTGGACGGAC-3′; adenosine receptor A1 (ADORA1), Forward 5′-CTGCCTGACTGTTCTGTCCA-3′, Reverse 5′-GCCTGTTCTGAATCCCAGAG-3′; adenosine receptor A2a (ADORA2a), Forward 5′-GATGGCAATGATGCCCTTAG-3′, Reverse 5′-TCCATCTTCAGTCTCCTGGC-3′; adenosine receptor A2b (ADORA2b), Forward 5′-TCCCCGTGACCAAACTTTTA-3′, Reverse 5′-TGACTTCTACGGCTGCCTCT-3′; adenosine receptor A3 (ADORA3), Forward 5′-CAGTTTCATGTTCCAGCCAA-3′, Reverse 5′-ATCGCTGTGGACCGATACTT-3′; actB, Forward 5′-GTTGTCGACGACGAGCG-3′, Reverse 5′-GCACAGAGCCTCGCCTT-3′.

### Purine Enzyme Uptake Assay

Magnetic bead purified naïve B cells were resuspended in RPMI 1640 culture media (without serum) to a final density of 5 × 10^7^ cells/mL, and warmed to 37°C. To 20 μL of cell suspension was added 2.5 μL of RPMI or 2.5 μL of recombinant human CD73 (prepared in RPMI at 1:500 from manufacturer’s stock), then 2.5 μL of 500 μM ^14^C-AMP (Moravek Biochemicals). After gentle mixing, the suspension was returned to a heating plate at 37°C (final concentration of 4 × 10^7^ cells/mL and 50 μM ^14^C-AMP). After 3 min of incubation, samples were centrifuged at 1000× *g* for 1 min. Ten microliters of supernatant were added to a microtube containing 30 μL of a solution containing 50 mM EDTA (Gibco), and 30 μM EHNA (ADA1 inhibitor, Tocris), in PBS (Invitrogen) and this volume was frozen at −20°C for subsequent thin-layer liquid chromatography. The remaining supernatant was transferred to a glass vial, and 20 μL of RPMI was added gently to the cell pellet that was quickly centrifuged at 1000× *g* for 1 min. The supernatant was transferred to the original supernatant glass vial for that sample, then the cell pellet was resuspended in 20 μL of fresh RPMI and the cells were transferred to a new glass vial. Six milliliters of liquid scintillation cocktail (Opti-Fluor, Perkin Elmer) were added to the glass vials, which were mixed thoroughly before analyzing C-14 counts per minute (CPM) on a Packard Bioscience Tri-Carb 2100TR liquid scintillation analyzer.

### Thin-Layer Chromatography

Controls (^14^C-AMP, ^14^C-Ado, and ^14^C-inosine – Moravek Biochemicals) and samples were applied to silica gel matrix thin-layer chromatography plates (Sigma-Aldrich) and migrated until the solvent (6:3:1 2-propanol:ammonium hydroxide:D H_2_0) front reached 4 cm beyond the application site. Plates were dried and incubated in a storage phosphor screen cassette overnight, after which the screens were analyzed on a GE Storm 860 Imager. Scanned images were evaluated using the ImageJ software package gel analysis tools (version 1.49, Wayne Rasband, National Institutes of Health, USA) to quantify densitometry relative to a dilution series of ^14^C-Ado run on the same plates as experimental samples.

### Statistical Analysis

Statistical tests described in the figure legends were performed using GraphPad Prism version 5.0b for Mac.

## Results

Previous studies by our group and others had raised the possibility that compared to circulating total adult B cells (all CD19^+^ cells), total newborn B cells have distinct (lower) expression of both CD73 and CD39 (19) and lower AMPase activity (17). As adult circulating B cells include a large number of activated and memory B cells, and newborns do not have significant numbers of these cells, we sought to evaluate the expression of CD73, CD39, and other receptors or enzymes involved in the generation of Ado or Ado signaling on subsets of B cells in circulation from human adult and newborn study participants. Gating strategies for evaluating or isolating subsets of B cells are shown in Figure 1, and have been previously described (18).

**Figure 2:**
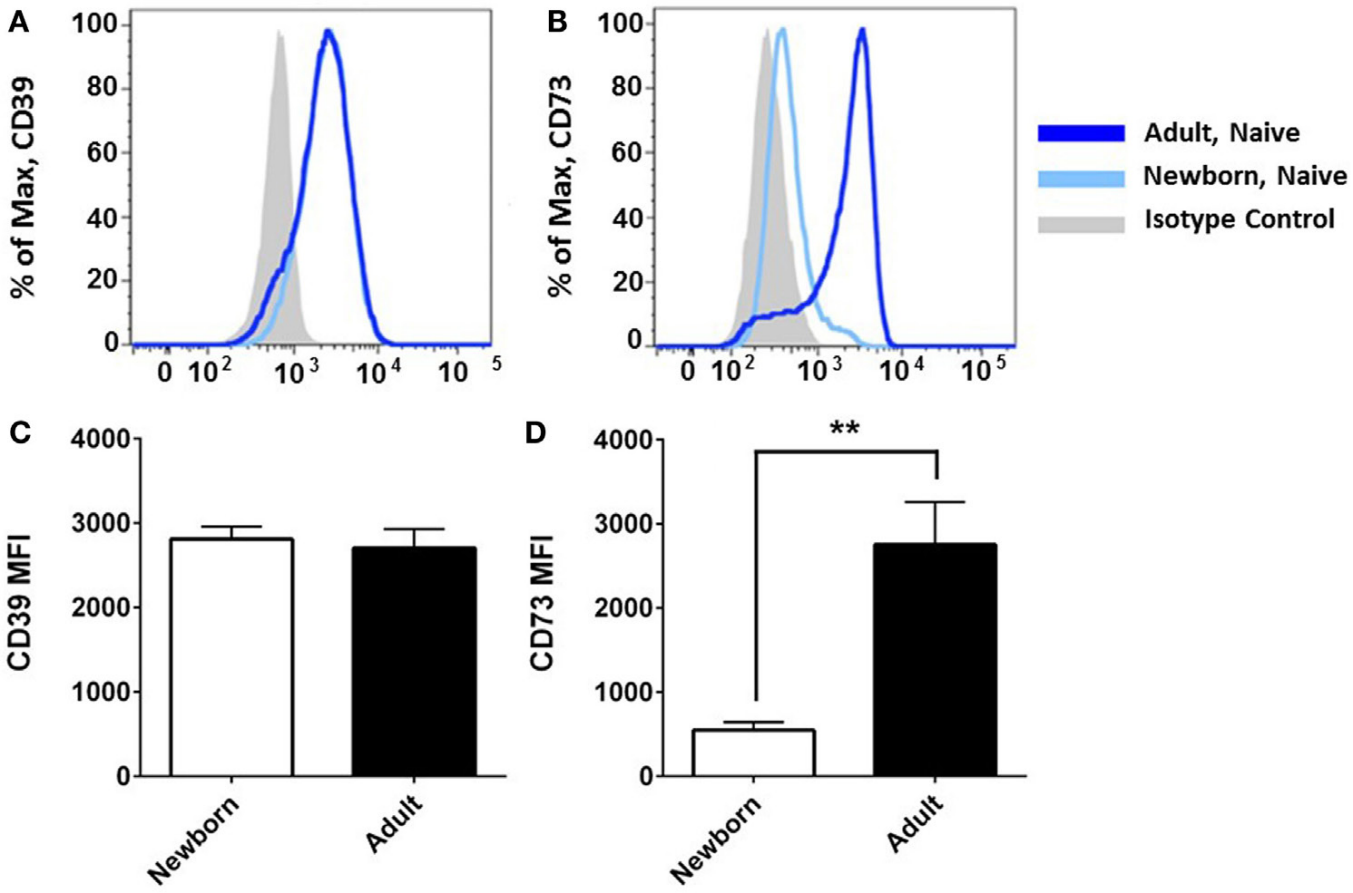
**Neonatal naïve B cells have a significant deficiency of CD73 surface expression**. Flow cytometry analysis reveals that CD39 surface expression was similar on naïve B cells from neonatal cord blood or adult peripheral blood **(A,C)**, but CD73 is strikingly deficient on neonatal naïve B cells **(B,D)**. Data shown in **(A,B)** are from one independent experiment, representative of *N* = 5–9 subjects for each target, respectively. **(C,D)** present mean fluorescent intensity (MFI) for *N* = 5 newborn subjects and 9 adult subjects, bars represent population means and error lines are the standard error mean (SEM), **p < 0.01, two-tailed Student’s *t*-test.

**Figure 3:**
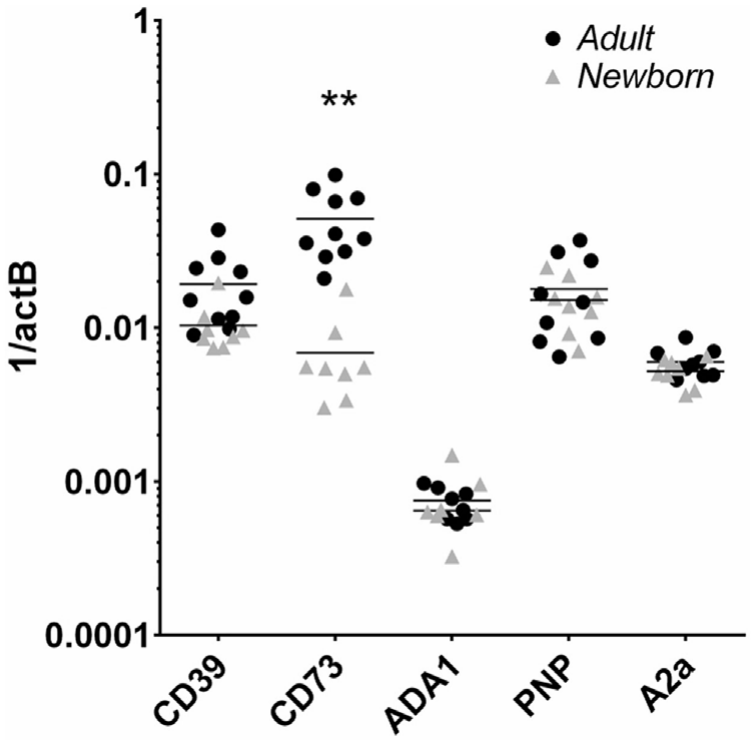
**Human neonatal naïve B cells are selectively deficient in CD73 mRNA compared to adult counterparts**. Adult and newborn circulating naïve B cells were isolated by FACS and evaluated for mRNA expression of purine enzymes CD39, CD73, ADA1, PNP, and the Ado receptor A2a. *N* = 8 (neonatal) or 10 (adult), horizontal lines represent the mean, **p < 0.01, two-tailed Student’s *t*-test.

Flow cytometry revealed that CD39 surface expression was similar on circulating nave B cells from adults or newborns (Figures 2A,C), but that CD73 expression was strikingly lower on circulating neonatal naïve B cells (Figures 2B,D). Deficiency of CD73 expression on newborn naïve B cells was similar on transitional naïve B cells (CD24^++^) and mature naïve B cells (CD24^+^, data not shown). We also evaluated mRNA expression of the primary enzymes involved in extracellular Ado generation (CD39, CD73, TNAP), enzymes involved in intra- and extracellular purine nucleoside metabolism (ADA1, PNP), and all four receptors for extracellular Ado receptors (A1, A2a, A2b, and A3), in subsets of circulating B cells from adults and newborns. Cells were sorted by FACS to isolate pure populations of naïve B cells (CD19^+^IgD^+^CD27^−^) from newborn cord blood samples, and naïve IgD^+^ memory (CD19^+^IgD^+^CD27^+^), class-switched memory (CD19^+^IgD^−^CD27^+^CD38^±^), and plasmablasts (CD 9^+^IgD^−^CD27^+^CD38^++^), from adult peripheral blood. Very low signal for a minority of samples or no signal at all was detected for TNAP or Ado receptors A1, A2b, or A3 (data not shown). mRNAs encoding ADA1 and PNP were consistently detected at similar levels between newborn and adult naïve B cells. The only Ado receptor for which mRNA was consistently detected was the A2a receptor that was similarly expressed in newborns and adult naïve B cells. CD39 mRNA was similar between newborn and adult naïve B cells, while CD73 mRNA was significantly lower on newborn naïve B cells (Figure 3), as had been observed for surface protein.

**Figure 4:**
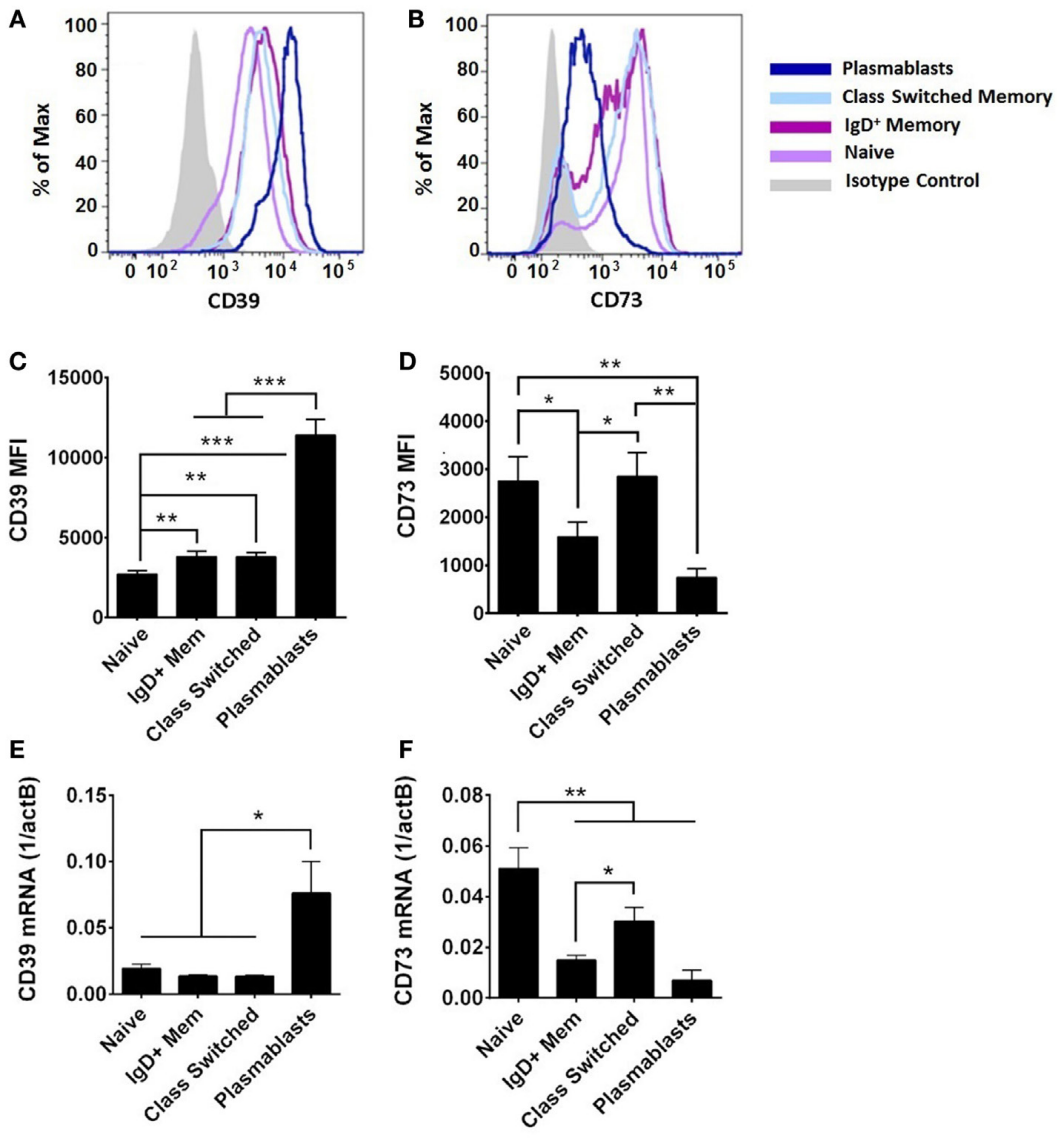
**CD39 and CD73 expression varies on different subsets of adult B cells**. Surface protein was evaluated by flow cytometry [CD39 in **(A,C)**, CD73 in **(B,D)**] and mRNA from FACS-isolated B cell subsets was evaluated by reverse transcription and qPCR **(E,F)**. **(A,B)** are representative of, and **(C,D)** quantitative analysis of, *N* = 9 adult subjects, statistical analysis repeated measures ANOVA with Holm–Sidak multiple comparison correction. **(E,F)** *N* = 5–9 (nine adults evaluated, insufficient numbers of plasmablasts for analysis of some samples). Statistical analysis by paired Student’s *t*-test, bars represent population means and error lines are standard error means (SEM), *p < 0.05, **p < 0.01, ***p < 0.001.

On adult circulating B cells, CD39 expression was elevated with markers of further activation and differentiation (Figures 4A,C), being higher on CD27^+^ B cells than on naïve B cells, and highest on plasmablasts (IgD^−^CD24^−^CD27^+^CD38^++^). By contrast, CD73 expression was highest on naïve and class-switched circulating B cells, with subpopulation of IgD^+^ memory and class-switched cells having diminished surface expression of CD73, and plasmablasts having consistently low expression (Figures 4B,D). CD39 and CD73 expression was similar on transitional B cells (IgD^+^CD24^++^CD27^−^CD38^++^) and naïve counterparts for both newborn and adult subjects (data not shown). CD39 and CD73 mRNA expression in subsets of adult circulating B cells correlated with detection of surface protein by flow cytometry (Figure 4). CD39 was most highly expressed on plasmablasts (Figures 4C,E). CD73 expression is regulated with B cell maturation, with the highest levels on naïve cells, and consistent reduction of mRNA and protein on IgD^+^ memory cells. Class-switch recombined memory B cells had similar levels of CD73 expression to naïve cells, whereas plasmablasts demonstrated very low levels of both mRNA and protein expression for CD73 (Figures 4D,F). mRNA expression of purine enzymes ADA1 and PNP was also detected in all subsets of adult B cells, but differences were not statistically significant in other subsets relative to adult naïve B cells (data not shown).

**Figure 5:**
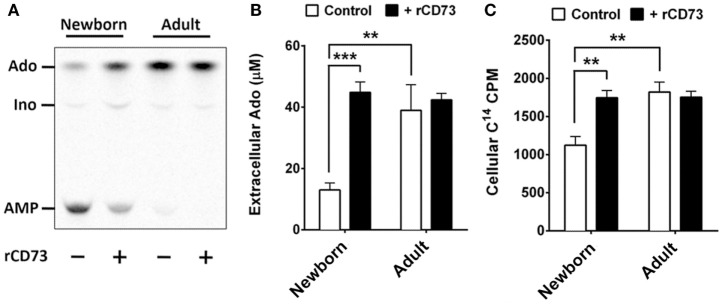
**Neonatal naïve B cells demonstrate lower levels of CD73-mediated Ado generation, and subsequently lower levels of Ado uptake**. **(A)** Isolated neonatal and adult naïve B cells were incubated with radio-labeled adenosine mono-phosphate (C^14^-AMP) to observe the rate of CD73-mediated adenosine generation. Purine composition of the extracellular supernatant was evaluated by thin-layer chromatography, and cellular uptake of radio-labeled purine nucleosides was measured in cell fractions using liquid scintillation. Positions for AMP, Ado, and Inosine (Ino) were determined using radiolabeled controls. **(A)** is 1 representative of four to six thin-layer chromatographs of extracellular fluid, **(B)** is quantitative analysis of chromatographs for *N* = 6 newborn and *N* = 4 adult subjects. **(C)** is quantitative analysis of C^14^ purine uptake into cell fractions for *N* = 6 newborn and *N* = 4 adult subjects. Impaired neonatal AMP de-phosphorylation and purine acquisition can be overcome by the addition of exogenous recombinant CD73 protein. Statistical analysis by Student’s *t*-test, unpaired for newborn vs. adult, paired for newborn control vs. newborn with rCD73, bars represent population means and error lines are standard error means (SEM), **p < 0.01, ***p < 0.001.

All human cells express equilibrative and concentrative nucleoside transporters (ENTs and CNTs, respectively) capable of acquiring purine nucleosides from the extracellular environment, whereas charged nucleotides are not transported across cell membranes. Extracellular purines have important signaling functions but can also be utilized for purine salvage if they can be acquired as nucleosides. Extracellular adenine nucleotides must be completely dephosphorylated prior to acquisition. While CD39 is expressed on both newborn and adult naïve B cells at similar levels, CD73 deficiency on newborn naïve B cells may limit the purine acquisition rate from the extracellular environment for purine salvage. We tested this by isolating naïve B cells from newborn cord blood and adult peripheral blood using magnetic bead isolation, and exposing them to radio-labeled AMP (C^14^) to evaluate the de-phosphorylation of AMP, and additionally to evaluate the cellular acquisition the generated C^14^-Ado. Adult naïve B cells dephosphorylated nearly all of the AMP within 3 min, which could not be increased by addition exogenous recombinant human CD73 (rCD73), whereas newborn naïve B cells had a lower rate of AMPase activity that could be enhanced considerably by addition of exogenous rCD73 enzyme (Figures 5A,B). At baseline, the rate of C^14^-Ado acquisition by newborn naïve B cells was significantly lower than that for adult counterparts (Figure 5C). The difference in purine acquisition was due to deficiency of CD73 expression on the newborn naïve B cells, as addition of exogenous enzyme increased their C^14^-Ado acquisition to adult-like levels (Figure 5C). Thus, neonatal deficiency of naïve B cell CD73 expression functionally limits acquisition of extracellular purines.

## Discussion

Neonatal circulating naïve B cells have a significant and selective deficiency of the purine enzyme CD73 relative to adult counterparts, which we have demonstrated at the level of mRNA expression, surface protein expression, and enzyme activity. After entry to the circulation, expression of CD73 and CD39 is also regulated during stages of maturation of B cells potentially impacting B cell activity.

Our results represent the most thorough characterization of human newborn B cell purine metabolism to date and suggest significant and selective differences between human and murine B cells in stage-specific expression of CD73. In mice, CD73 is expressed primarily on B cells that have undergone class-switch recombination (12), and is used as marker of memory (13, 14), whereas our data indicate that among circulating human B cells CD73 is expressed at the highest levels on naïve cells, with moderate reductions in expression on IgD^+^ memory cells. Murine germinal center B cells express high levels of CD73 (15), whereas in humans CD73 is expressed on very few B cells in germinal centers (20). These species-specific differences highlighting the importance of assessing findings from animal model systems in primary human samples.

In addition to differences in mRNA and protein expression, we also demonstrated for the first time that neonatal naïve B cell CD73 deficiency significantly impaired their capacity to acquire extracellular purines for purine salvage. Charged nucleotides are not transported across cell membranes; but after complete de-phosphorylation, purine and pyrimidine nucleosides can be acquired from the extracellular environment. The de-phosphorylation of AMP to generate the purine nucleoside Ado is primarily mediated by the enzymes CD73 or TNAP, of which B cells express CD73. Purine salvage is considerably more energy efficient than *de novo* purine synthesis and, thus, acquiring extracellular purine nucleosides may be critical during lymphocyte expansion. Activated T cells rapidly up-regulate glucose acquisition systems to meet energy demands (21), and lymphocyte activation and clonal expansion require increased biosynthesis of nucleic acids and other cellular building blocks (22). Incorporation and purine salvage of extracellular Ado by resting lymphocytes, and to a much greater degree in proliferating lymphocytes, have previously been demonstrated (23), and purine enzyme activity may be rate-limiting in the generation of accessible purine nucleosides for purine salvage. Future studies will evaluate the contribution of CD73 to the energy status and biosynthetic capacity of lymphocytes via purine nucleoside mobilization.

While CD73 has been best characterized in relation to purine metabolism, it is also an adhesion molecule during lymphocyte trafficking. CD73 expression mediates interactions between B cells and follicular dendritic cells in germinal centers (20). In mice, CD73^−/−^ lymphatic vessels supported normal migration of CD73^+/+^ lymphocytes, whereas CD73^−/−^ lymphocytes were significantly impaired in reaching lymphoid tissues via CD73^+/+^ lymphatic vessels (24). Accordingly, B cell stage-specific expression of CD73 may influence B cell trafficking in addition to influencing purine metabolism and purinergic signaling. If so, neonatal B cell deficiency in CD73 might limit migration of neonatal B cells to lymphoid tissues.

In prior studies, we have noted that human newborn blood plasma contains relatively high AMPase activity, partially due to soluble CD73 (sCD73) and TNAP that contribute to high plasma Ado levels at birth (19, 25). Whether the higher sCD73 and TNAP concentrations in newborn plasma may partially compensate for the lower B cell-associated CD73 may depend on the extent to which plasma proteins enter lymph nodes, a topic of future studies. Deficiency of CD73 in newborn naïve B cells may be due to differences inherent to the environment in which they develop relative mature adult naïve B cells, which would interact to a more considerable degree with memory T cells in lymphoid tissues. Additionally, many extracellular factors that influence immune cell function and development are detected at significantly different levels in newborn and adult subjects (26).

**Figure 6:**
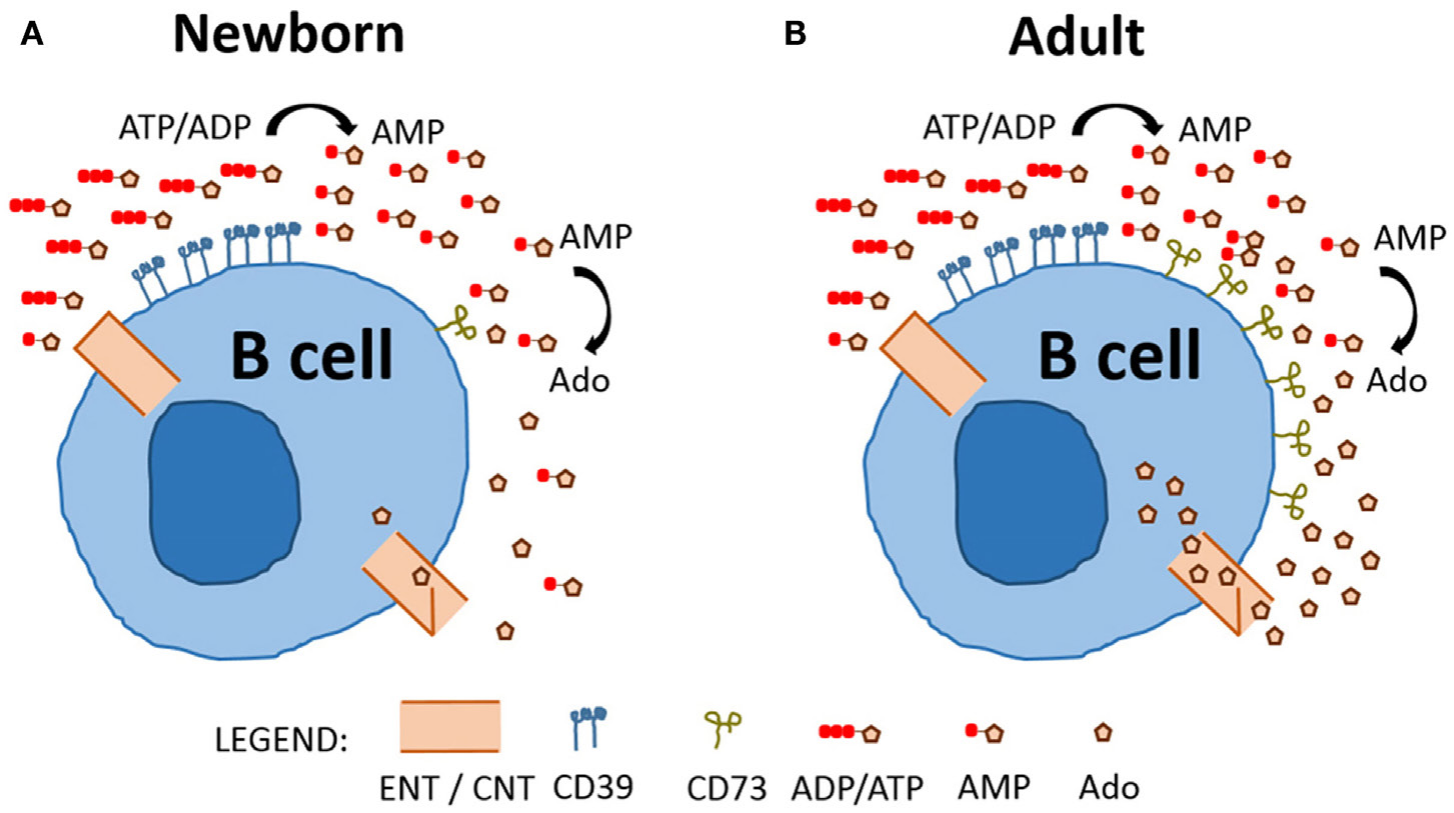
**Model comparing neonatal and adult naïve B cell purine metabolism**. Charged purine nucleotides are not acquired via nucleoside transporters (equilibrative nucleoside transporters, ENTs) or concentrative nucleoside transporters (CNTs) and require de-phosphorylation prior to uptake of nucleosides. Newborn **(A)** and Adult **(B)** naïve B cells express similar levels of CD39 and dephosphorylate ADP/ATP to generate AMP, but newborn naïve B cells have low expression of CD73 and, therefore, a reduced capacity to dephosphorylate AMP to generate the nucleoside Ado which can be internalized.

Overall, our study represents a fresh characterization of purine enzyme expression on circulating human B cells, revealing selective and functionally relevant differences in CD73 expression between newborn (Figure 6A) and adult B cells (Figure 6B). Deficient expression of CD73 on neonatal B cells impacts purine acquisition by limiting Ado generation and transport into B cells for purine salvage. It will be important for future studies to evaluate the impact of impaired purine salvage capacity in neonatal B cell populations on specific B cell functions, such as class-switch recombination, antibody production, and proliferation. Distinct features of neonatal B cell purine metabolism may contribute to the known impairment of neonatal B cell responses to infection and immunization (27). Accordingly, selective modulation of purine pathways might enable optimization of vaccines to be administered in early life (28).

## Author Contributions

MP designed and conducted the experiments and wrote the manuscript. OL provided overall mentorship and assisted in writing the manuscript.

## Conflict of Interest Statement

The authors declare that the research was conducted in the absence of any commercial or financial relationships that could be construed as a potential conflict of interest.
